# Hydrotropic Solubilization by Urea Derivatives: A Molecular Dynamics Simulation Study

**DOI:** 10.1155/2013/791370

**Published:** 2013-02-21

**Authors:** Yong Cui

**Affiliations:** Small Molecule Pharmaceutical Development, Genentech Inc., 1 DNA Way, South San Francisco, CA 94080, USA

## Abstract

Hydrotropy is a phenomenon where the presence of a large quantity of one solute enhances the solubility of another solute. The mechanism of this phenomenon remains a topic of debate. This study employed molecular dynamics simulation to investigate the hydrotropic mechanism of a series of urea derivatives, that is, urea (UR), methylurea (MU), ethylurea (EU), and butylurea (BU). A poorly water-soluble compound, nifedipine (NF), was used as the model solute that was solubilized. Structural, dynamic, and energetic changes upon equilibration were analyzed to supply insights to the solubilization mechanism. The study demonstrated that NF and urea derivatives underwent significant nonstoichiometric molecular aggregation in the aqueous solution, a result consistent with the self-aggregation of urea derivatives under the same conditions. The analysis of hydrogen bonding and energy changes revealed that the aggregation was driven by the partial restoration of normal water structure. The energetic data also suggested that the promoted solubilization of NF is favored in the presence of urea derivatives. While the solutes aggregated to a varying degree, the systems were still in single-phase liquid state as attested by their active dynamics.

## 1. Introduction

Enhancing aqueous solubility of poorlysoluble drugs is crucial to the success of drug formulations [1]. Hydrotropic solubilization, the technique whereby the addition of one solute significantly promotes the solubility of another [2], has been widely applied to this end [3–5]. Yet, the nature and mechanisms of hydrotropic solubilization remain elusive and a topic of debate [6], especially with regard to the structural diversity of hydrotropic agents (HAs) and the apparent drug-hydrotrope selectivity [7–9]. This situation, in combination with the compelling application needs, demands more structure-specific understanding of the hydrotropic effect. In fact, dating back to almost the beginning of the discovery of hydrotropy, Neuberg [2] already noted that the hydrotropic agents consisted generally of an anionic (hydrophilic) group and a hydrophobic aromatic ring or ring system, where the type of anion or metal ion appeared to have a minor effect. The aromatic ring, on the other hand, appeared to be critical to the hydrotropic effect. Molecular orbital calculation later suggested that the aromatic ring or ring system may facilitate a *π*-donor *π*-acceptor interaction [10, 11], a potential mechanism once widely believed to account for the hydrotropic effect of nicotinamide and other heteroaromatic hydrotropic agents [12]. Rasool et al. [13] also showed that aromatic ring systems may promote the stacking of molecules and, as a result, enhanced drug aqueous solubility more effectively than the aliphatic amides (e.g., urea and its derivatives). These evidences were in support of the classical molecular complexation mechanism, whereby the aromaticity of hydrotropic agents apparently played a critical role. Nevertheless, experimental findings inconsistent with this potential mechanism were also reported. For example, nicotinamide was shown to solubilize drugs in the absence of a *π*-donor *π*-acceptor interaction [6]. It was also found that the hydrophobicity, rather than the aromaticity, of hydrotropic agents is the determinant of their solubilization ability [7, 14, 15]. Clearly, these seemingly contradictory observations are essential in clarifying the mechanism of hydrotropy and therefore warrant further investigation.

In the light of this situation, we initiated a series of studies using molecular dynamics (MD) simulation, a technique with an atomic-level resolution, to probe hydrotropic solubilization. In an earlier study [16], we successfully modeled the solubilization of a poorly water-soluble anti-HIV compound, PG-300995, by the hydrotropic agent nicotinamide. The study clearly showed that the solubilization was driven by the restoration of normal water structure that was disrupted by the dispersion of solutes. This process was accompanied by significant molecular aggregation of nicotinamide and the drug in an aqueous solution. The *π*-donor *π*-acceptor interaction and the so-induced molecular complexation were not the determinant of the solubilization. In a second study [17] on the solubilization of riboflavin in the presence of caffeine, the parallel stacking of the solutes was found. Despite that the solute clusters present different appearances for these two systems, the driving forces are the same: the restoration of normal water structure. With these interesting findings, the current study attempted to further expand the MD simulation to aliphatic amides, another group of hydrotropic agents. The purposes of this study are to elucidate the hydrotropic solubilization mechanism of aliphatic amides and, in combination with our earlier reports, to provide a more comprehensive understanding to the mechanism of hydrotropy.

**Figure 1:**
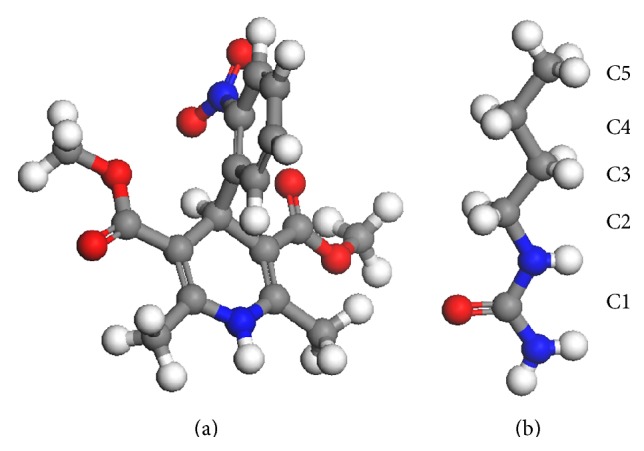
Molecular configurations of nifedipine (a) and butylurea (b).

The study employed model systems consisting of nifedipine (NF), a poorlysoluble antihypertension drug, solubilized by a series of urea analogues in an aqueous environment. Hydrotropic solubilization of NF was previously reported [15] in the presence of urea (UR), methylurea (MU), ethylurea (EU), and butylurea (BU), respectively, with the solubilization effect ranked as BU > EU > MU > UR (see Figure 1 for chemical structures of NF and butylurea). A similar progressive solubilization effect correlated with increasing hydrophobicity was also found in some other hydrotropic agents [7]. Therefore, studying such model systems may also help elucidate the role of hydrophobicity in hydrotropic solubilization.

## 2. Computational Method

The solubility of NF (MW 346.3) in water (pH 7) is approximately 5-6 µg/mL [18], or approximately 0.17 × 10^−4^ M. It was reported that the solubility of NF had approximately 3-, 15-, 65-, and 65-folds increase in the presence of UR (4 M), MU (4 M), EU (4 M), and BU (1.2 M), respectively (estimated from Figure  6 of [15]). These results translate to NF solubilities below 0.2 : 1000 (NF : water, molar ratio) in the presence of these HAs, whose concentrations are approximately 72 : 1000 (HA : water) for UR, MU, and EU (4 M), or 22 : 1000 for BU (1.2 M). Given these experimental results and bearing in mind the limited computation resource, the number of water molecules was held at 1000 in all model systems. Four primary models were constructed containing (1) one NF and 72 UR in 1000 water (NF + UR + Water system) (2) one NF and 72 MU in 1000 water (NF + MU + Water system) (3) one NF and 72 EU in 1000 water (NF + EU + Water system) and (4) one NF and 37 BU in 1000 water (NF + BU + Water system). As such, the concentrations for HAs were 4 M for UR, MU, and EU and 2 M for BU (all in solution state based on experimental solubility data [7]). The BU concentration in the NF + BU + Water system was adjusted to approximately 2 M from 1.2 M in the experimental system. The NF was supersaturated in these models but was accepted as this study was designed to elucidate the qualitative mechanism rather than to quantify the solubility.

To study behaviors of HAs in aqueous solutions in the absence of NF, four additional models were built: (1) 72 UR in 1000 water (UR + Water system) (2) 72 MU in 1000 water (MU + Water system) (3) 72 EU in 1000 water (EU + Water system) and (4) 37 BU in 1000 water (BU + Water system). Furthermore, several additional model systems were constructed in the same manner to assist the analysis of energetic and dynamic changes upon hydrotropic solubilization. Details are provided later in the text. In all models, solutes were randomly dispersed in the solvent (1000 water molecules) and all molecules were treated unionized in accordance to the experimental condition of pH 7.

Model systems were set up using the Amorphous Cell module of the Material Studio (MS) 4.3 software package. Cubic simulation boxes with periodic boundary conditions in all directions were constructed with a density of 1 g/cm^3^, followed by the geometric optimization of molecular configurations utilizing COMPASS force field. COMPASS is a Class II *ab initio* force field designed for use with organic molecules and optimized for the simulation of condensed phases (see [19–21] for the parameterization and validation of this force field). Figure 1 shows the optimized molecular configurations of NF and BU. The MD simulations were then carried out under NPT conditions in the Forcite module of MS, with pressure and temperature held at 100 kPa and 298 K, respectively, by employing Nose thermostat and Berendsen barostat. The COMPASS force field was used to calculate both van der Waals and electrostatic interactions. Charge groups were applied with cutoff distances of 14 Å for both interactions (among the eight models given above, the smallest simulation box size after MD runs is >33 Å. A box containing 1000 water molecules has a size >30.5 Å). The time step was 1 fs, and the simulation time was at least 3 ns. For most models, simulations were run up to 4 ns, and successful equilibration was confirmed. For further details of the simulation method, readers are referred to the work of Sellner et al. [22] and the references therein.

Data analysis was performed using the Forcite Analysis function. For calculating radial distribution functions (RDFs), the amide carbon atom was selected to represent HA molecules as the amide group is shared by all HAs. The oxygen atom was chosen for water molecules as this is the only heavy atom in water. The calculation cutoff distance and step size were set at 25 and 0.1 Å, respectively. The RDFs at the starting time were calculated by averaging over the first 100 ps of the simulations and denoted as *t* = 0 ns in the text, while those of the ending time were averaged over the last 1 ns of the runs and denoted as *t* = 3 ns. For hydrogen bond calculations, a cutoff distance of 2.5 Å was applied. The total number and the average length of HBs in the systems were averaged over the first five (5 ps) and the last 20 (20 ps) frames with a step size of 1 ps. Since the initial states of the systems equilibrated rapidly, the total number of HBs was constantly increasing, which led to large variations in the averaged values even in the first 20 ps. Thus, only the first 5 ps were used to calculate the initial states. In calculating mean square displacements, molecular centroids were defined, and the calculation was conducted with a step size of 1 ps and a calculation length of 500 ps over the last 1 ns period.

## 3. Results and Discussion

### 3.1. Visual Inspection

**Figure 2:**
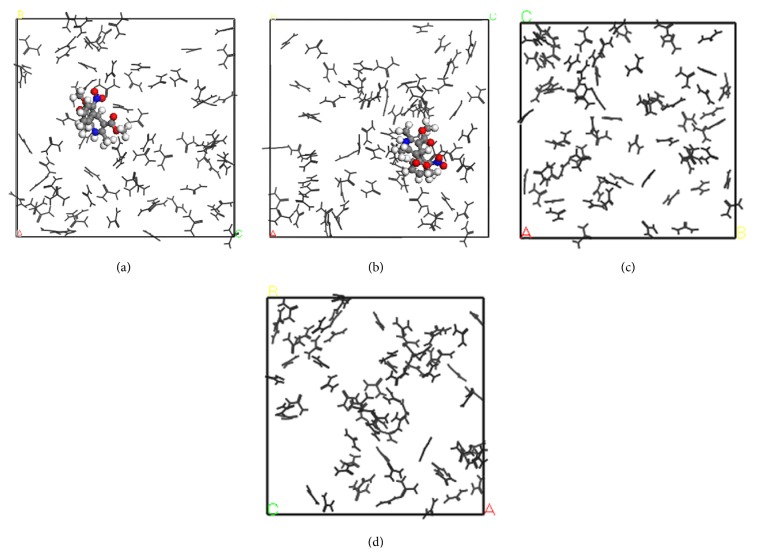
Snapshots of MD simulations of urea aqueous solutions with and without nifedipine. Water molecules are deleted for the sake of clarity. The nifedipine molecule is in colored ball and stick, and urea molecules are in black line. (a) One nifedipine and 72 urea in 1000 water molecules (NF + UR + Water system), *t* = 0 ns; (b) NF + UR + Water system, *t* = 3 ns; (c) 72 urea in 1000 water molecules (UR + Water system), *t* = 0 ns; (d) UR + Water system, *t* = 3 ns.

**Figure 3:**
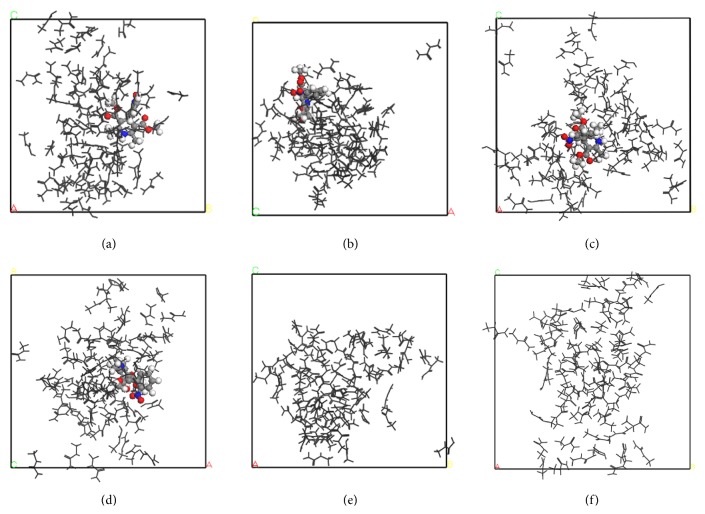
Snapshots of MD simulations of methylurea aqueous solutions with and without nifedipine. Water molecules are deleted for the sake of clarity. The nifedipine molecule is in colored ball and stick, and methylurea molecules are in black line. (a–d) NF + MU + Water system. (a) *t* = 3 ns, OA direction; (b) *t* = 3 ns, OC direction; (c) *t* = 4 ns, OA direction; (d) *t* = 4 ns, OC direction. (e and f) MU + Water system, OA direction. (e) *t* = 3 ns; (f) *t* = 4 ns. OA, OB, and OC are the three dimensions of the cubic simulation boxes; O is the original point.

**Figure 4:**
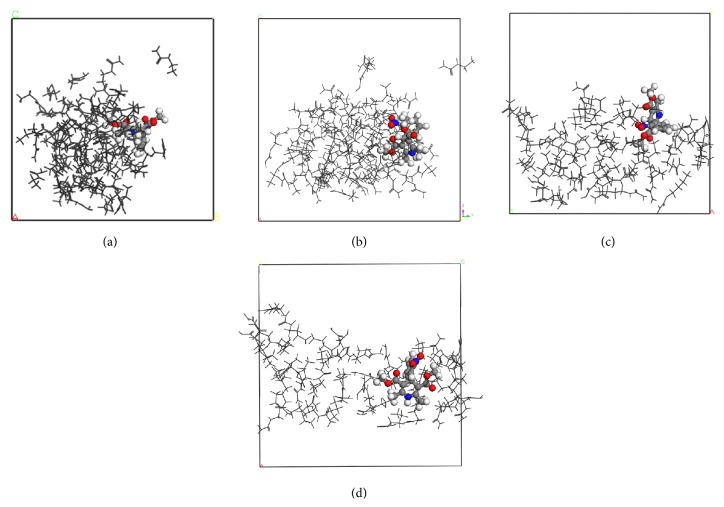
Snapshots of MD simulations of ethylurea and butylurea aqueous solutions with nifedipine. Water molecules are deleted for the sake of clarity. The nifedipine molecule is in colored ball and stick, and ethylurea molecules are in black line. All frames were viewed from OA direction. (a and b) NF + EU + Water system, *t* = 3 ns (a) and *t* = 4 ns (b). (c and d) NF + BU + Water system, *t* = 3 ns (c) and *t* = 4 ns (d).

The snapshots of simulated systems are provided in Figures 2–4. The representativeness of these snapshots was checked by examining the last 0.5 ns of each running trajectory. Both NF + UR + Water and UR + Water systems in Figure 2 appeared to be homogeneous solutions after the 3 ns simulation, with little structural changes perceived visually. Clearly, these two systems were still one-phase systems after the NPT runs. The NF + MU + Water and MU + Water systems, on the other hand, demonstrated significant aggregation of solute molecules after 3 ns as shown in Figures 3(a)-3(b) and Figure 3(e), respectively. Furthermore, in comparing frames captured at 3 (Figures 3(a), 3(b), and 3(e)) and 4 ns (Figures 3(c), 3(d), and 3(f)) from the same perspectives, drastic changes in the shape of aggregates were observed for both NF + MU + Water and MU + Water systems over the period of 1 ns, indicating that the aggregates were rather fluidic. While clearly in the aggregation mode, the translational movement of MU molecules swept across the whole simulation box in 1 ns, showing clearly a single-phase liquid system. Similar to the MU systems, significant aggregation of solutes was also observed in the NF + EU + Water (Figures 4(a)-4(b)) and the NF + BU + Water (Figures 4(c)-4(d)). The snapshots of the EU + Water and BU + Water systems were omitted due to their resemblance to Figure 4. When comparing frames of the EU and BU systems at 3 (Figures 4(a) and 4(c)) and 4 ns (Figures 4(b) and 4(d)), morphological changes of aggregates were also evident, albeit that solutes in these systems appeared to be in a more clearly defined “aggregation” mode constantly in 1 ns, in contrast to the UR and MU systems. Consequently, for the BU and EU systems, it is difficult to tell by visual inspection in 1 ns period whether the aggregates are still in a single phase with aqueous medium or phase-separated. Finally, in all models simulated, the aggregation was found nonstoichiometric, and little parallel stacking was perceived among the solute molecules.

The observation above supplies an interesting insight to the potential of phase separation. In the case of MU systems in particular, snapshots at 3 or 4 ns alone all show clearly the aggregates. It is difficult to tell just by the snapshots at a certain time point whether the systems are phase-separated. Combining together the snapshots of multiple time points, that is, 3 and 4 ns, however, it is clear that the aggregates are quite volatile and are actively mixing with the solvent medium, indicating that these aggregates are still miscible with the solvent. In other words, the HA molecules appear as aggregates when the observation time is short, that is, in fs, while they are miscible solutions when observed over a much longer time frame. This hints that, by a conventional experimental observation, whose time frame is typically 1–100 s, these aggregates could be perceived visually as miscible with the solvent media (as one-phase solutions). Therefore, it seems that the potential phase separation issue may be attributed to the drastically different observation times between simulation studies and conventional experimental observations. This reconciles the apparently conflicting conclusions obtained from the visual observation and the simulation. Note that although the same conclusion is not as readily drawn for the EU and BU systems, the short simulation time (between 3 and 4 ns) relative to slower dynamics of EU and BU is likely to account for this vagueness.

### 3.2. Structural Changes

**Figure 5:**
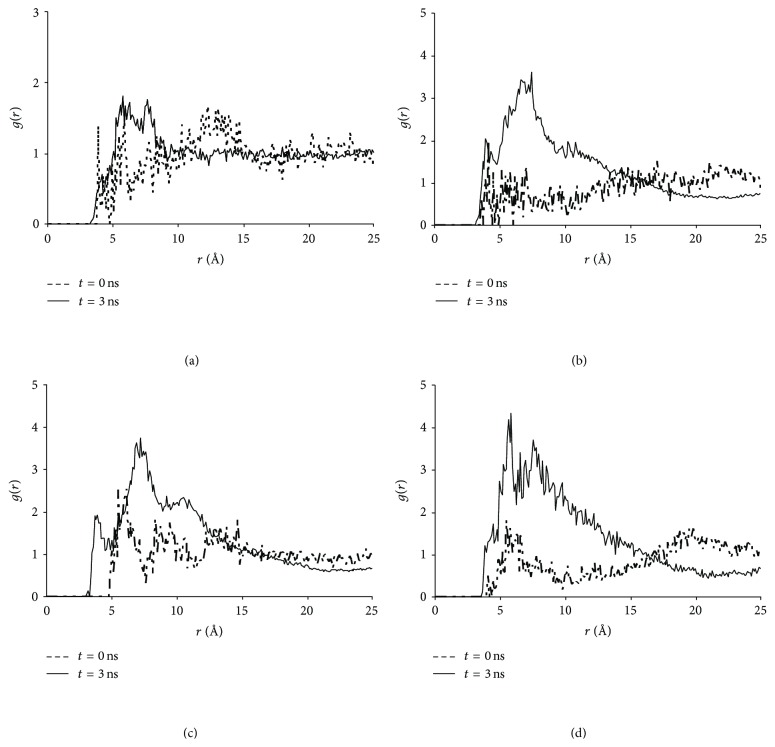
Nifedipine-urea derivative radial distribution functions in four hydrotropic solutions. (a) NF + UR + Water system; (b) NF + MU + Water system; (c) NF + EU + Water system; (d) NF + BU + Water system.

**Figure 6:**
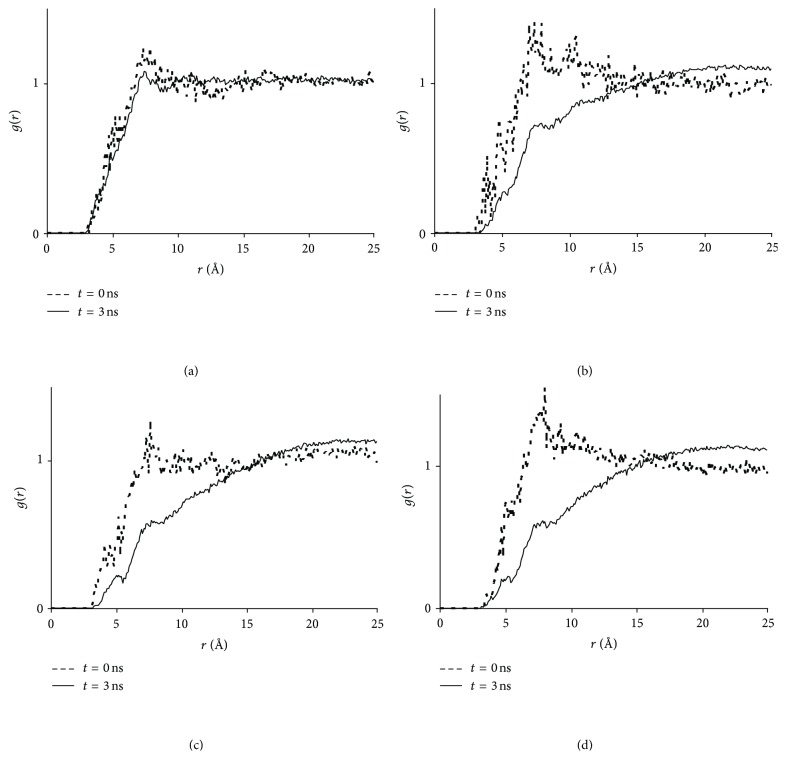
Nifedipine-water radial distribution functions in four hydrotropic solutions. (a) NF + UR + Water system; (b) NF + MU + Water system; (c) NF + EU + Water system; (d) NF + BU + Water system.

We further examined structural evolutions of the simulated solutions by inspecting radial distribution functions (RDFs) within and between each type of molecules.  Figure 5 depicts RDFs between NF and four hydrotropic agents as averaged over the periods of 0–100 ps (*t* = 0) and 2-3 ns (*t* = 3 ns), respectively. The NF-UR RDFs in Figure 5(a) show that the peak shifted to a shorter distance and was slightly strengthened after the simulation, indicating a weak but positive aggregation of the NF and UR molecules. This effect was too minor to be visually perceived from the snapshots in Figure 2. Additionally, Figures 5(b)–5(d) record clear and significant peak strengthening towards closer distances for the remaining three hydrotropic solutions after 3 ns. Furthermore, the NF-Water RDFs for the four hydrotropic solutions are given in Figure 6. Except for the NF + UR + Water system (Figure 5(a)), which exhibits a fairly minor change, the remaining three (Figures 5(b)–5(d)) all show significant declines in water concentration in the neighborhood of the NF molecule after 3 ns, suggesting that the aggregation reduces the exposure of NF to the solvent. This is consistent with the visual observations presented earlier and confirms that significant aggregations took place in these solutions.

**Figure 7:**
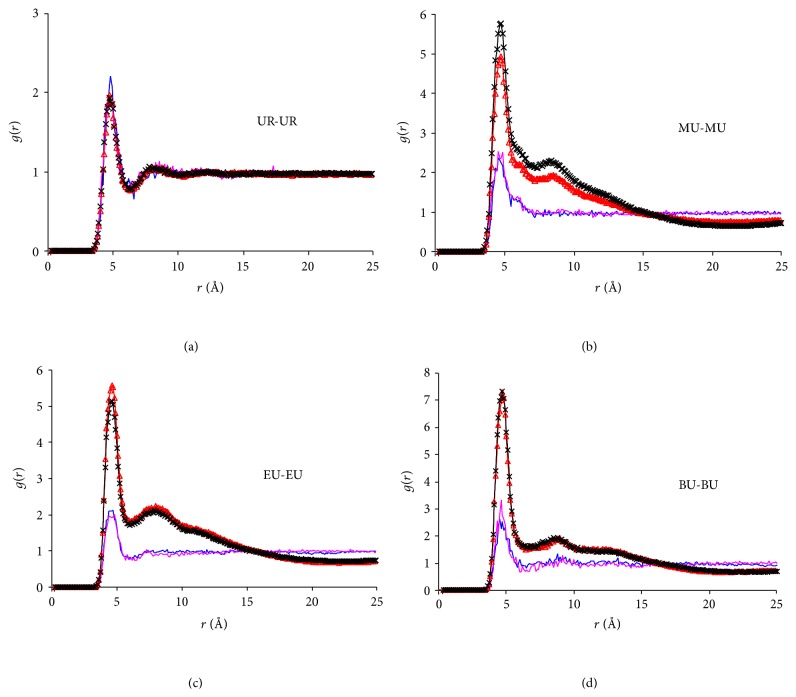
Radial distribution functions between urea derivatives. (a) UR-UR RDFs. Red open triangle: NF + UR + Water system, *t* = 3 ns; black cross: UR + Water system, *t* = 3 ns; blue line: NF + UR + Water system, *t* = 0 ns; purple line: UR + Water system, *t* = 0 ns; (b) MU-MU RDFs. Red open triangle: NF + MU + Water system, *t* = 3 ns; black cross: MU + Water system, *t* = 3 ns; blue line: NF + MU + Water system, *t* = 0 ns; purple line: MU + Water system, *t* = 0 ns; (c) EU-EU RDFs. Red open triangle: NF + EU + Water system, *t* = 3 ns; black cross: EU + Water system, *t* = 3 ns; blue line: NF + EU + Water system, *t* = 0 ns; purple line: EU + Water system, *t* = 0 ns; (d) BU-BU RDFs. Red open triangle: NF + BU + Water system, *t* = 3 ns; black cross: BU + Water system, *t* = 3 ns; blue line: NF + BU + Water system, *t* = 0 ns; purple line: BU + Water system, *t* = 0 ns.

**Figure 8:**
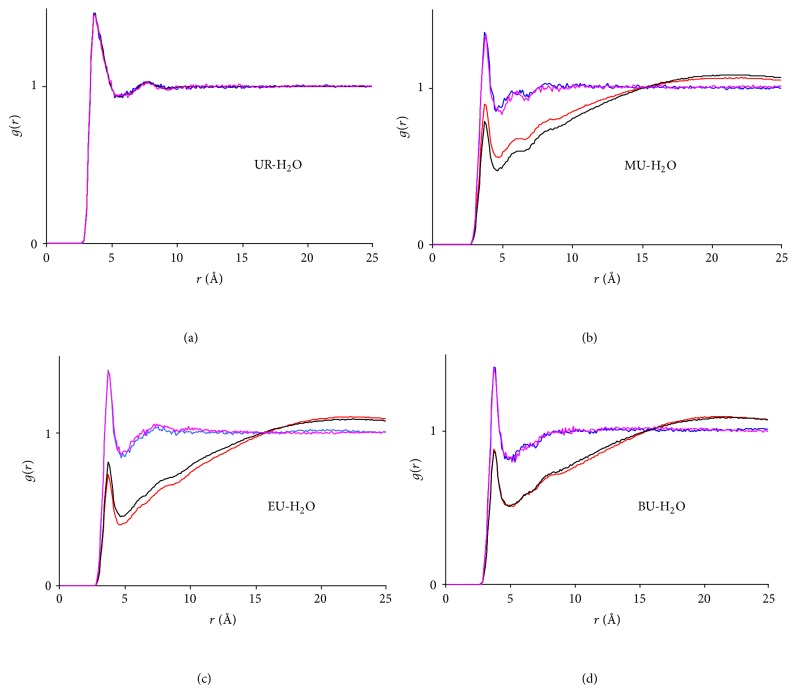
Radial distribution functions between hydrotropic agents and water. (a) UR-H_2_O RDFs. Red line: NF + UR + Water system, *t* = 3 ns; black line: UR + Water system, *t* = 3 ns; blue line: NF + UR + Water system, *t* = 0 ns; purple line: UR + Water system, *t* = 0 ns; red and black lines overlapped with blue and purple lines; (b) MU-H_2_O RDFs. Red: NF + MU + Water system, *t* = 3 ns; black: MU + Water system, *t* = 3 ns; blue: NF + MU + Water system, *t* = 0 ns; purple: MU + Water system, *t* = 0 ns; (c) EU-H_2_O RDFs. Red: NF + EU + Water system, *t* = 3 ns; black: EU + Water system, *t* = 3 ns; blue: NF + EU + Water system, *t* = 0 ns; purple: EU + Water system, *t* = 0 ns; (d) BU-H_2_O RDFs. Red: NF + BU + Water system, *t* = 3 ns; black: BU + Water system, *t* = 3 ns; blue: NF + BU + Water system, *t* = 0 ns; purple: BU + Water system, *t* = 0 ns.

The comparison between the structures of the hydrotropic solutions with and without NF was conducted via the HA-HA and HA-H_2_O RDFs as shown in Figures 7 and 8, respectively. Again, the UR-UR (Figure 7(a)) and UR-H_2_O (Figure 8(a)) RDFs in both NF + UR + Water and UR + Water systems exhibited little changes after the simulations, while substantial increases in HA concentrations and decreases in water concentration near the HA molecules were revealed for the remaining six systems (Figures 7(b)–7(d) and 8(b)–8(d)). This confirms significant aggregations taking place in these systems. More importantly, the HA-HA and the HA-H_2_O RDFs of the four hydrotropic solutions (with NF) resemble closely their respective counterparts of the four HA solutions (without NF), suggesting that structurally the systems in the presence of NF correlate closely with their counterparts in the absence of NF. This advocates that the self-aggregation of HAs may be a prerequisite in enabling the incorporation of the drug into the HA aggregates. Interestingly, earlier reports [23–25] indicated that hydrotropic agents often tend to self-aggregate in aqueous solutions, which is consistent with the results above.

**Figure 9:**
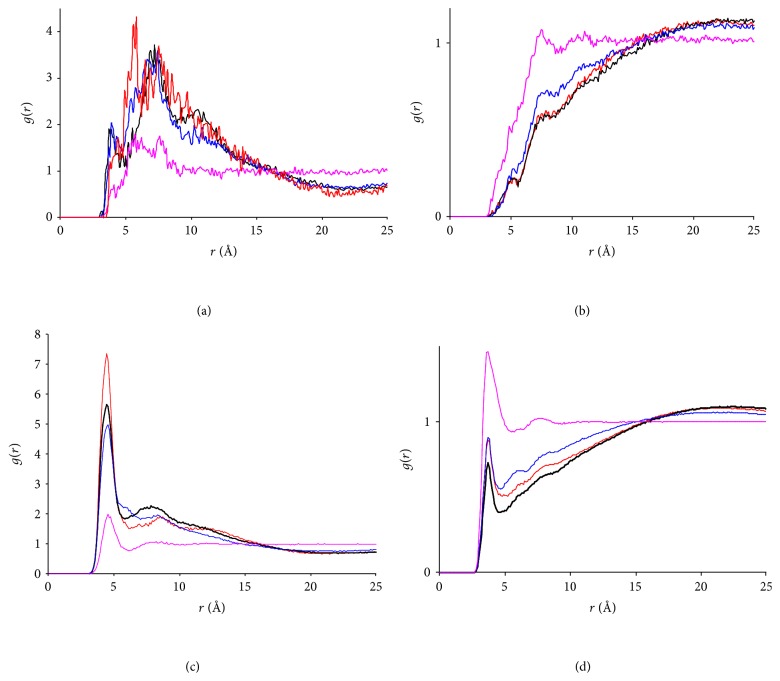
The overlay of RDFs in four hydrotropic agents at *t* = 3 ns. (a) NF-HA RDFs; (b) NF-H_2_O RDFs; (c) HA-HA RDFs; (d) HA-Water RDFs. Red: NF + BU + Water system; black: NF + EU + Water system; blue: NF + MU + Water system; purple: NF + UR + Water system.

Figure 9 compares various RDFs across the four hydrotropic solutions (with NF) at *t* = 3 ns. It can be seen from Figures 9(a)–9(c) that the aggregation in these systems can be approximately ranked as BU > EU > MU > UR, indicating that the growing hydrophobicity of the HAs enhances their aggregation and reduces the water concentration in the neighborhood of the NF and HA molecule. This result is expected and can be readily accounted for by our current understanding on hydrophobic interactions. From the methodology perspective, it supplies a solid evidence validating the simulation approach adopted. It is worth further noting that the urea derivatives in the study vividly show a gradual transition from a true homogeneous solution to a state with solutes clustered together while it has yet been phase-separated. This characteristic possibly is a key factor giving rise to hydrotropic solubilization. Typically, solutions at concentrations lower than the compound solubility are in homogeneous state, while those at concentrations higher than the solubility are often phase-separated, either in experimental or in simulated studies. The urea derivatives seem to behave in the middle of these two ends. While their aggregation protects hydrophobic solutes from water, and therefore, the degree of aggregation correlates with the solubilization effect of these HAs (i.e., BU > EU > MU > UR) [15], refraining from phase-separation is crucial to maintain a single-phase liquid state.

**Figure 10:**
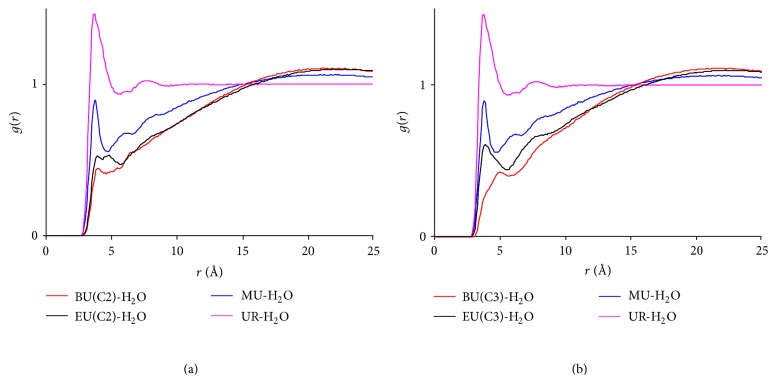
The overlay of RDFs between hydrotropic agents and water in four hydrotropic solutions at *t* = 3 ns. EU and BU were represented by C_2_ (a) and C_3_ (b) in the calculation. See Figure 1 for carbon atom numbering.

A second note is that the HA-H_2_O RDFs in Figure 9(d) show that the declines in water concentration near HA molecules are ranked as EU > BU > MU > UR, with the maximum decline claimed by EU instead of BU. The flipping over of the positions of EU and BU was unexpected. It was suspected that as the hydrophobic alkyl chains grow and the molecule elongates, the amide carbon atom sitting at the hydrophilic end of the molecule may become less representative for the whole HA in the RDF calculation. This was proved by the EU-H_2_O and BU-H_2_O RDFs recalculated using alternative carbons C_2_ and C_3_ (see Figure 1 for the numbering of carbons of BU. EU used the same numbering sequence) of EU and BU, which are illustrated in Figure 10. It is evident that water concentration near the alkyl chain (represented by C_2_ and C_3_) of BU is lower than that of EU. Hence, the results in general are consistent with those above and support that the degree of aggregation correlates positively with the growing hydrophobicity of HAs.

**Figure 11:**
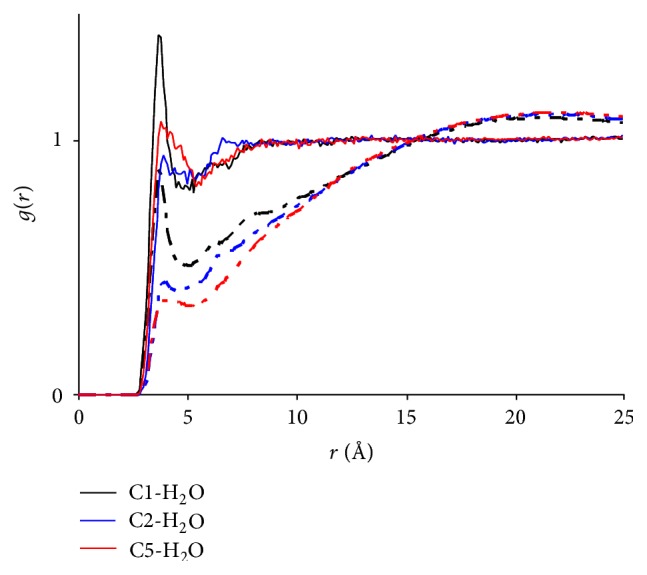
Radial distribution functions between water and different carbon atoms of butylurea in the NF + BU + Water system. Refer to Figure 1 for atom numbering. Solid lines: *t* = 100 ps; dashed lines: *t* = 3 ns.

The above finding hints that the distribution of water may be heterogeneous along the elongated HA molecules. To demonstrate this, time evolutions of RDFs between water and various carbons along BU are compared in Figure 11. It is clearly seen that while the water concentration around the amide carbon (C_1_) still tops the rest after 3 ns, those of C_5_ and C_2_ switch their places upon simulations with C_5_ becoming the one with least surrounding water. This evidence shows that during aggregation, BU molecules reorient themselves with their hydrophobic alkyl chains shying away from water, an effect similar to the micelle formation of amphiphilic agents in water. The difference is, though, that no apparent self-assembling of BU molecules is observed as witnessed in Figure 4. It, therefore, suggests that aside from micelle formation the solute aggregation may be another effect driven also by hydrophobic interactions and functioning similarly to micelles in promoting the solubility of hydrophobic compounds. A similar comparison (data not shown) between the water concentrations surrounding C_2_ and C_3_ of EU indicates that the water concentration around C_3_ is still slightly higher than that of C_2_. This can be attributed to the insufficient length of the alkyl chain of EU, whose hydrophobicity is not strong enough to drive the alkyl chain away from water. Overall, hydrophobic interactions appear to account for the aggregation of urea derivatives.

**Figure 12:**
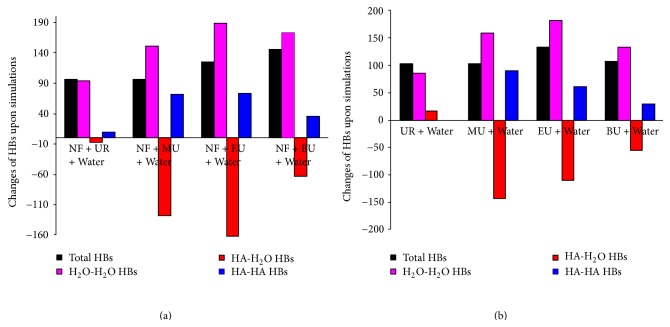
Changes of hydrogen bonds in eight urea analogue solutions upon simulations. (a) Hydrotropic solutions (with NF); (b) solutions of hydrotropic agents (without NF).

To confirm the role of hydrophobic interactions, we analyzed changes in hydrogen bonds (HBs) in these systems upon simulations. The results are summarized in Table 1 and Figure 12. A few observations are in order (1) all eight systems are dominated by H_2_O-H_2_O HBs (approximately 82–93% of the total HBs) both before and after simulations, followed by HA-H_2_O (approximately 4–17% of the total HBs) and HA-HA HBs (approximately 0.1–5% of the total HBs). The HBs sponsored by NF, that is, NF-HA and NF-H_2_O HBs, are trivial (<0.3% of the total HBs); (2) upon simulations, the numbers of H_2_O-H_2_O and HA-HA HBs increase in all systems, while those of HA-H_2_O HBs decrease except in the UR + Water system (see Figure 12). Again, changes in NF-HA and NF-H_2_O HBs are negligible, despite that NF-HA HBs consistently present slight increases as a result of the inclusion of NF in aggregations. The total numbers of HBs, however, exhibit appreciable increases in conjunction with the declines in HB bond length in all systems (see Table 1), indicating that HB interactions in these systems are strengthened upon simulations. Overall, these changes, that is, gains in H_2_O-H_2_O and HA-HA HBs in exchange of losses in HA-H_2_O HBs, are in agreement with the aggregations observed, whereby the partitioning of solutes and water should promote HB interactions between water molecules as well as between solute molecules but demote HBs across water and solutes. These exchanges result in significant net gains in the number and strength of HBs, which are obviously energetically favorable; (3) from the water structure perspective, it is interesting to compare the HBs in pure water with those in the eight solution systems in study. To this end, a system containing 1000 water molecules only (the Water system) was constructed and subjected to a 3 ns NPT run in the same manner as described in Section 2. Table 1 lists the number of H_2_O-H_2_O HBs of the Water system after simulation, which is appreciably higher than that of the eight solution systems in study. Additionally, the average HB bond length in the Water system is the shortest compared to that of the eight systems, indicating that H_2_O-H_2_O HBs are stronger than other types of HBs in these solutions. The evidences demonstrate that homogeneous dispersion of urea derivatives in water disrupts the water structure and reduces substantially the H_2_O-H_2_O HBs, while aggregations of solutes promote the energetically more favorable H_2_O-H_2_O HBs in these systems via a partial restoration of the normal water structure. This follows exactly how hydrophobic interactions take effect in water solutions. On this basis, we may attribute the restoration of the water structure, and thereby the aggregation of solutes, to hydrophobic interactions; (4) further analysis across various types of HBs shows (see Figure 12) that HA-H_2_O HBs score the largest changes (declines) upon simulations in all cases, while H_2_O-H_2_O HBs present slightly lower changes (increases). The changes in HA-H_2_O and H_2_O-H_2_O HBs are close in magnitude in some cases (e.g., the NF + MU + Water and NF + EU + Water systems), but opposite in direction. The HA-HA HBs, on the other hand, show some smaller but solid gains except in the two UR-containing systems (i.e., the UR + Water and NF + UR + Water systems). This result may suggest that self-interactions between urea derivatives may also play a role to the aggregation. Especially in cases where the declines in HA-H_2_O HBs are in magnitude comparable to the increases of the H_2_O-H_2_O HBs, favorable self-interactions between HA molecules may not be trivial. It should be noted that HA self-interactions are independent from hydrophobic interactions. Even if hydrophobic interactions alone may induce HA aggregations, which set the stage for HA self-interactions, they do not guarantee enhanced HA-HA HBs between HA molecules. The latter rely on the chemical natures of the HAs. Thus, the current results seem to suggest that the aggregations of urea derivatives can be accounted for by the coordinated effects of hydrophobic interactions and the self-interactions between hydrotropic agents.

### 3.3. Dynamic Changes

**Figure 13:**
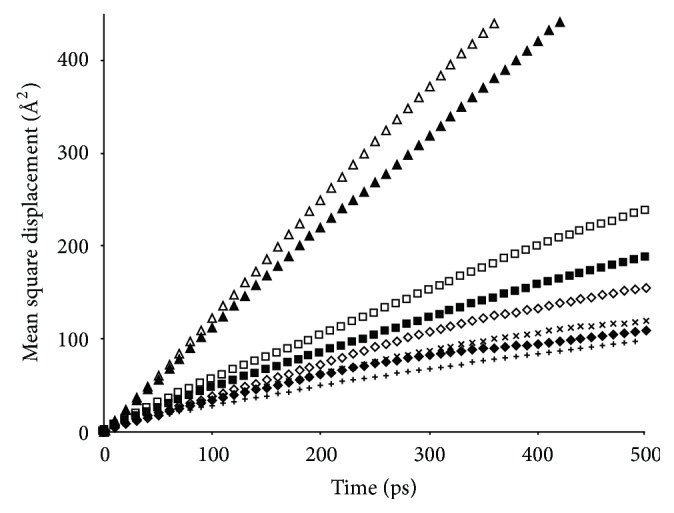
Mean square displacements of centers of hydrotropic agents between 2 and 3 ns. ⊗: UR in UR + Water system; ▲: UR in NF + UR + Water system; □: MU in MU + Water system; ■: MU in NF + MU + Water system; ⋄: EU in EU + Water system; *♦*: EU in NF + EU + Water system; ×: BU in BU + Water system; +: BU in NF + BU + Water system.

The mean square displacements (MSDs) of the HA molecules were calculated for the eight systems in study in the last 1 ns of the runs and are plotted in Figure 13. It is evident that the slopes of MSD-time curves are ranked as UR > MU > EU > BU. No plateau is observed for any system. The diffusion coefficients (*D*) derived from slopes of the curves via *D* = slope/6 are given in Table 2, which indicates clearly that the dynamics of the aggregates slow progressively as the HA hydrophobicity grows. The diffusion coefficients range from 0.31 to 2.1 (×10^−9^ m^2^/s), falling in the typical range of diffusion coefficients in liquids (the bulk data of diffusion coefficients in liquids appear in the magnitude of 10^−9^ –10^−10^ m^2^/s) [26–28]. This indicates that the aggregates in these systems are still in liquid state, a result consistent with the active dynamics discussed earlier. To differentiate these liquid states from pure amorphous phases of the HAs, diffusion coefficients of HAs in amorphous state were estimated under the same temperature and pressure. To do so, four amorphous systems containing 300 UR, MU, EU, and BU molecules, respectively, were constructed and subsequently subjected to NPT runs of 3 ns. The simulations were performed under the same conditions as described previously. The smallest box size (300 URs) of the four systems is 31 Å. The diffusion coefficients were then calculated in the same manner as shown above and are listed in Table 2. It is readily seen that the diffusion coefficients of HAs in amorphous state are about two orders of magnitude lower than the ones in aggregates, indicating that the aggregates are much more active dynamically than the amorphous phase of the respective HAs. This evidence is in favor of the case that the aggregates are not amorphous/liquid phases of HAs separated from water medium. More likely, as suggested earlier, the active dynamics of HA molecules may keep the aggregates from being phase-separated.

It should be noted that the presence of the NF molecule seems to reduce consistently slopes of MSD-time curves for all four HAs (see Figure 13 and Table 2), signifying that the addition of NF hinders the mobility of HAs in the aggregates. This is certainly different from the nicotinamide hydrotropic solution reported earlier [16], where virtually no impact on the mobility of the hydrotropic agent (nicotinamide) was found following the addition of the drug. It is somewhat surprising that one NF molecule can effectively slow the mobility of much large numbers of HA molecules, which apparently cannot be accounted for by just a handful of hydrogen bonds formed between the NF and HA molecules (see Table 1). We have not found an explanation for this observation.

To verify our simulation results, diffusion coefficients of HAs in dilute aqueous solutions were estimated by the Wilke and Chang method [26, 27], and the results are provided in Table 2. The estimated diffusion coefficients are of the same order of magnitude but about 1–3-folds higher than the values obtained from the simulations. Since the Wilke and Chang method only applies to dilute solutions, where solute-solute interactions are negligible, it is understandable that diffusion coefficients estimated by this method are somewhat higher than those in concentrated solutions. Furthermore, a clear trend is observed in Table 2 that the discrepancy between these two groups of diffusion coefficients grows larger as UR < MU < EU < BU, a trend correlating positively to the increasing degree of aggregation of the HAs. Thus, it seems reasonable to attribute the discrepancy in diffusion coefficient from these two sources to the impediment of molecular diffusion by the increasing degree of HA aggregation. Overall, the simulated diffusion coefficients in solutions appear reasonable relative to the empirically estimated values.

### 3.4. Energetic Changes

To explore energy changes upon aggregation, another system was built containing one NF in 1000 water (PG + Water system) and was subjected to a 3 ns NPT run under the same conditions as described above. The average total energy of this system during the last 1 ns was normalized to per mole of water and is listed in Table 3, together with that of eight systems in study and of the Water system (1000 water molecules). Applying the formula proposed by Sellner et al., the energy gain of the hydrotropic solubilization, that is, of incorporating the NF into the HA aggregates, can be estimated as Δ*E*_solubilization_ = (*E*_NF+HA+Water_ − *E*_HA+Water_)−(*E*_NF+Water_ − *E*_Water_). The two brackets on the right side of the equation represent sequentially energy changes upon the addition of NF to the HA + Water and to the Water systems. The difference between these two energy changes, Δ*E*_solubilization_, is negative when the solubility of NF in the HA + Water systems is higher than that in the Water system, provided the changes in entropy and volume upon adding one NF are comparable between the two systems [16]. The more negativity of Δ*E*_solubilization_ indicates a more enhanced solubility of NF. Table 3 shows that Δ*E*_solubilization_ for various HAs are all negative, that is, −0.500 (UA), −0.517 (MU), −0.596 (EU), and −0.607 (BU) kJ/mol water, indicating that hydrotropic solubilization is energetically favored in all four HA solutions. The magnitudes of Δ*E*_solubilization_ are ranked as BU > EU > MU > UA, corresponding to the solubilization effects measured experimentally on these systems (i.e., approximately 3-, 15-, 65-, and 65-folds increase in NF solubility in UR [4 M], MU [4 M], EU [4 M], and BU [1.2 M] solutions [15]). Note that the BU concentration was raised to 2 M in our simulations, and thereby a higher degree of solubilization in the BU solution was expected, which seemed to be reflected in the highest Δ*E*_solubilization_ value of the BU system. Nevertheless, given the large standard deviations of these average total system energies as listed in Table 3, the differences in Δ*E*_solubilization_ between various HA systems are by no means statistically significant. The large standard deviation is an inherent weakness of this calculation procedure, where relatively large values (average total system energies) are subtracted leaving the results (differences) submerged in the background noises (standard deviations) [22]. To conduct an accurate calculation, significantly longer simulations are required to reduce noises (e.g., [22] employed simulations of 30 ns). We extended the simulations to 4 ns and found that the improvements were minimal. Restricted by computation resources and considering also that the goal of this study is more of mechanistic elucidation than of quantitative energy calculation, further extension of simulations was not pursued. Setting aside the specific ranking, the negative Δ*E*_solubilization_ values indeed show that the hydrotropic solubilization of NF is energetically favored in all urea derivative solutions.

To summarize, the study finds nonstoichiometric molecular aggregation of urea derivatives in their aqueous solutions. The degree of aggregation correlates positively with the hydrophobicity of urea derivatives. Further analysis shows that partial restoration of normal water structure drives the nonstoichiometric molecular aggregation, despite that self-interactions between HA molecules have also contributed to this process. The results can be accounted for by our current understanding on hydrophobic interactions. Furthermore, energetic data support that the hydrotropic solubilization of NF is favored in the presence of urea derivatives in aqueous solutions.

It is particularly interesting to note that urea derivatives demonstrate a gradual transition from a homogeneous solution to a highly aggregated state. These transition states feature active solute dynamic movements, which give rise to a single-phase liquid state in spite of a varying degree of solute aggregation. We speculate that this characteristic could be the key for certain agents to display hydrotropic solubilization effects in aqueous solutions.

In the spirit of the discussion above, we may reflect on the size of aggregates and the aggregation number. Historically, the aggregation (or complexation) number, the number of molecules involved in one aggregate, was a critical parameter. Based upon the molecular complexation hypothesis [13, 29, 30], or alternatively, upon the molecular aggregation hypothesis [6, 31], this number was often estimated by fitting the experimental solubility data to appropriate kinetic functions and was used in return to support the respective hypothesis. In either way, the value obtained was low, with the maximum reported value of 4.37 [31]. This is obviously inconsistent with our observations in simulation studies, which appeared far beyond a handful. Yet, as shown in the case of MU, the aggregates are completely miscible with solvent media if given sufficient observation time. This apparently disqualifies a classical concept of static aggregation. That is, despite that the HA molecules are in aggregated mode within a short period of time, for example, 1 ps, the active translational movements render the aggregates very volatile and of very short shelf lives. Therefore, with one aggregate constantly re-dispersed/restructured in the solvent medium, the number of solute molecules incorporated in such an “aggregate” is not set, and thereby the aggregation number or the size of the aggregate under the classical view of an “aggregate” may not be relevant, unless the observation time is shorter than the life span of these dynamic “aggregates.”

## 4. Conclusion

This study finds a varying level of nonstoichiometric molecular aggregation of urea derivatives in their aqueous solutions. Structural and dynamic evidences show that the degree of aggregation correlates positively with the hydrophobicity of urea derivatives. Analysis of HB changes upon simulations provides insights that strengthening HB interactions as a result of the restoration of normal water structure facilitates the aggregation and solubilization of the model drug. This study, in combination with our earlier reports [16, 17], demonstrates that the restoration of normal water structure is the ultimate driving force for hydrotropic solubilization, despite that solute clusters may take different formats. This mechanism is in accordance with hydrophobic interactions, the underlying driving force for the micelle formation of amphiphilic solutes. Hence, maybe it is fair to say that solutes may cluster, as a result of hydrophobic interactions, into various states including aggregates, parallel stacks, and micelles. All these clusters may have the potential to incorporate hydrophobic solutes and consequently increase their solubility in water. Hence, despite that hydrotropic agents do not undergo self-assembly as surfactants in solutions, hydrophobicity still plays a fundamental role in inducing hydrophobic interactions and, therefore, is critical to the solubilization effects. Aromatic ring structures, on the other hand, induce the same effect of hydrophobic interactions, as shown in our earlier reports [16, 17], without involving the hydrophobic alkyl chains. In other words, the underlying driving forces for urea derivatives, nicotinamide, and caffeine are the same, despite their different structural characteristics.

## Figures and Tables

**Table 1:** Hydrogen bonds in the four hydrotropic solutions (with NF) and the four hydrotropic agent solutions (without NF).

	Time^a^	HA–HA^b^	HA–H_2_O	NF–HA	NF–H_2_O	H_2_O–H_2_O	Total number of H-bonds ± SD	Average length of H-bonds ± SD, Å
NF + UR + Water	Starting	14 ± 5	348 ± 8	1 ± 1	4 ± 2	1660 ± 14	2027 ± 15	2.0013 ± 0.0182
	Ending	23 ± 4	341 ± 8	1 ± 1	4 ± 1	1754 ± 14	2123 ± 20	1.9740 ± 0.0051
UR + Water	Starting	20 ± 3	327 ± 14			1683 ± 31	2030 ± 35	2.0040 ± 0.0173
	Ending	20 ± 3	343 ± 14			1769 ± 15	2133 ± 17	1.9776 ± 0.0052
NF + MU + Water	Starting	21 ± 4	295 ± 16	0	4 ± 1	1682 ± 32	2002 ± 27	2.0125 ± 0.0228
	Ending	93 ± 4	167 ± 11	4 ± 2	2 ± 1	1833 ± 21	2099 ± 27	1.9830 ± 0.0065
MU + Water	Starting	17 ± 4	298 ± 17			1687 ± 37	2002 ± 25	2.0152 ± 0.0240
	Ending	106 ± 8	155 ± 11			1845 ± 23	2105 ± 23	1.9867 ± 0.0055
NF + EU + Water	Starting	21 ± 3	281 ± 13	0	5 ± 1	1648 ± 28	1955 ± 31	2.0119 ± 0.0126
	Ending	94 ± 9	120 ± 10	1 ± 1	5 ± 1	1836 ± 20	2080 ± 21	1.9850 ± 0.0054
EU + Water	Starting	22 ± 5	287 ± 14			1652 ± 22	1961 ± 17	2.0111 ± 0.0148
	Ending	83 ± 5	178 ± 12			1834 ± 16	2094 ± 16	1.9829 ± 0.0063
NF + BU + Water	Starting	5 ± 1	155 ± 23	0	5 ± 1	1698 ± 40	1863 ± 49	2.0083 ± 0.0173
	Ending	41 ± 4	92 ± 8	3 ± 1	2 ± 1	1871 ± 18	2009 ± 16	1.9811 ± 0.0042
BU + Water	Starting	9 ± 2	150 ± 27			1728 ± 34	1887 ± 40	2.0143 ± 0.0175
	Ending	38 ± 5	95 ± 5			1861 ± 24	1994 ± 25	1.9788 ± 0.0053
Water	Ending					1964 ± 19		1.9712 ± 0.0065

^a^The HBs were averaged over the first 5 and the last 20 ps of each simulations.

^b^HAs: hydrotropic agents, that is, UR, MU, EU, and BU, respectively.

**Table 2:** Diffusion coefficients (× 10^−9^ m^2^/s) of HA in water solutions and amorphous states.

Hydrotropic agents		Diffusion coefficient of HA in solutions by simulation	Diffusion coefficient of HA in solution by Wilke-Chang estimation	Diffusion coefficient of HA in amorphous state by simulation
UR	UR + Water NF + UR + Water	2.018 1.721	2.395	0.011
MU	MU + Water NF + MU + Water	0.790 0.615	1.317	0.005
EU	EU + Water NF + EU + Water	0.525 0349	1.125	0.002
BU	BU + Water NF + BU + Water	0.396 0.315	0.962	0.002

**Table 3:** Energies of hydrotropic solubilization in four hydrotropic systems.

	NF + UR + Water	UR + Water	NF + MU + Water	MU + Water	NF + EU + Water	EU + Water	NF + BU + Water	BU + Water	NF + Water	Water
Average total energy (2-3 ns), kJ/mol water	−74.455	−74.382	−59.01 7	−58.927	−63.206	−63.037	−42.794	−42.614	−24.427	−24.854
SD	0.312	0.302	0.325	0.326	0.353	0.338	0.325	0.342	0.273	0.286

Δ*E* ^*^, kJ/mol water	−0.500		−0.517		−0.596		−0.607			
SD	0.293		0302		0.313		0.306			

^*^Δ*E* = Δ*E*_aggreagation_ = *E*_NF+HA+water_ − *E*_HA+water_ − *E*_NF+water_ + *E*_water_, where Has are hydrotropic agents, that is, UR, MU, EU, and BU, respectively.
